# Reasoning About Life Transitions in Relation to Perceived Housing: In-Depth Data From Sweden

**DOI:** 10.1093/geroni/igab046.1717

**Published:** 2021-12-17

**Authors:** Erik Eriksson, Steven Schmidt, Maya Kylén

**Affiliations:** 1 Lund University, Lund, Skane Lan, Sweden; 2 CASE Lund University, Lund, Skane Lan, Sweden

## Abstract

The accessibility and suitability of housing to improve health and well-being for the growing share of older adults in the population has important policy implications. Yet, current housing policies tend to neglect the heterogeneity of older adults housing needs which vary across age, health, and personal preferences. Little is known about how life transitions in combination with perceived housing relates to good aging. This qualitative study aims to explore the relationship between perceived housing, life transitions, well-being, health, and participation in older age. Participants were community-dwelling and aged 65-75 years. All participants reported multiple transitions considered as important for the way they viewed their housing situation and social relationships as well as for the choices they made around their housing. The findings from this project may be used to develop health promotion programs that proactively support housing decisions along the process of ageing and enable full participation in society.

